# Development of a scale assessing Beliefs About ThirdHand Smoke (BATHS)

**DOI:** 10.1186/s12971-017-0112-4

**Published:** 2017-01-17

**Authors:** Regine Haardörfer, Carla J. Berg, Cam Escoffery, Łucja T. Bundy, Melbourne Hovell, Michelle C. Kegler

**Affiliations:** 1 Department of Behavioral Sciences and Health Education, Rollins School of Public Health, Emory University, 1518 Clifton Rd NE, Atlanta, GA 30322 USA; 2 Center for Behavioral Epidemiology and Community Health, Graduate School of Public Health, San Diego State University, 9245 Sky Park Court, San Diego, CA 92182 USA

**Keywords:** Thirdhand smoke, Scale development, Secondhand/environmental exposure

## Abstract

**Background:**

Similarly to secondhand smoke (SHS), thirdhand smoke (THS) beliefs may be correlated with smoking behaviors and smokefree policies in the home. Thus, there is a need to develop and validate measures to assess beliefs about THS.

**Methods:**

A list of 19 items related to THS were generated by an expert panel and tested in a pilot study. Based on results from an exploratory factor analysis, two factors emerged: THS persistence in the environment and THS impact on health. The scale was reduced to nine items, which showed no differential item functioning by smoking status or smoking ban status in the home. The nine items and the two factor structure were tested in a validation sample from a smoke-free homes intervention that included THS educational materials.

**Results:**

The 9-item scale showed excellent internal consistency. Confirmatory factor analysis indicated good model fit for the two factor solution in a low-income population. Tests of construct validity indicated differences due to exposure to the smoke-free homes intervention, by smoking status, whether participants own or rent their home, and smoking ban status in the home.

**Conclusions:**

The BATHS scale offers researchers a valid and reliable tool to assess THS beliefs.

**Electronic supplementary material:**

The online version of this article (doi:10.1186/s12971-017-0112-4) contains supplementary material, which is available to authorized users.

## Background

Thirdhand smoke (THS) is a recent discovery that contributes to indoor pollution and compromises health [1]. The 3 R definition of THS describe it as the residual pollutant that **remains** on a variety of indoor surfaces and in dust, is **re-emitted** back into a gas phase, and **reacts** with other compounds in the air [2]. THS exposure may occur long after secondhand smoke appears (SHS) [3]. It can linger on surfaces long after cigarettes have been extinguished [4–6]. Matt (2011) [7] found that even weeks and months after a cigarette has been smoked, harmful particulates remain on countertops, floors, upholstery, carpets, clothing, and other surfaces. Furthermore, removal of nicotine residues from carpet and walls has been found to be nearly impossible [8], leading to continual exposure to THS. In addition, when the nicotine in tobacco smoke sorbed to indoor surfaces reacts with nitrous acid, a common component of indoor air pollutant, substantial levels of hazardous carcinogens called tobacco-specific nitrosamines (TSNAs) were formed, including NNA and NNK [5, 7, 9, 10]. NNA is absent in freshly emitted SHS, but a main TSNA formed in this process. In a recent study, Bo et al. demonstrated for the first time that exposure to THS, acute or chronic, increased DNA damage (genotoxicity) in human cell lines, which could lead to formation of cancer. While research on human exposure to THS and its effects on health, behavior and social cultural consequences warrants further study [6, 8–12], THS poses a likely health hazard to non-smokers who are exposed.

Infants and small children are especially susceptible to THS exposure because of their immature respiratory and immune systems and likelihood to crawl and play on, inhale, touch and hand-to-mouth contaminated surfaces, such as floors and upholstery [11, 12]. There also has been research that suggests that THS is potentially hazardous to the health of fetuses [13]. The poor are also more likely to be exposed to THS because smoking and SHS are more prevalent in low-income households. A recent study found the nicotine concentrations in the dust are higher in low-income non-smoking households than those with income above the median [14]. Matt et al.’s study [7] indicated that THS accumulates in smokers’ homes and persists even long after the smokers move out and the homes are cleaned and repainted for new residents. Non-smokers living in homes (houses, condos, apartments) formerly occupied by smokers are involuntarily exposed to THS [7]. Therefore, those with incomes that do not allow much free choice of rental housing might be more likely to be exposed to THS [15].

Knowledge and beliefs about SHS are correlated with smoking cessation and reduction; however, few studies have examined similar constructs about THS and how they may impact preventive smoking-related behaviors. Two studies have found no to limited awareness about THS compared to SHS [16, 17], while one of them showed that beliefs about THS’ harm on children was independently associated with home smoking bans [17]. Recently, education about THS has been incorporated into interventions to promote home smoking bans [18, 19]. Drehmer [20] found that THS harm beliefs were related to more strict enforcement of smoke free bans in homes and cars and increased numbers of quit attempts, which is encouraging evidence for inclusion of THS education in interventions aiming to decrease the impact of tobacco use [21]. Based on this literature, more accurate knowledge and beliefs about THS might be associated with being a nonsmoker, having a smoke-free home, and having been exposed to education regarding the harms of THS.

A reliable and valid scale of THS beliefs might enable more precise assessment of the belief that THS is harmful. It may enable assessment of the degree to which such beliefs contribute to smoke-free home bans, avoidance of SHS in homes and other indoor spaces. The purpose of this article is to present the development of a THS Beliefs Scale and its initial psychometric properties tested among participants of a survey panel.

## Methods

### Participants & procedures

#### Pilot sample

Data for an exploratory factor analysis came from a larger study evaluating a website aimed at helping people establish home smoking bans to make their home smoke-free. Participants were recruited in 2014 via email from Lightspeed GMI, a double-opt in access panel with panel participants from across the United States. Eligibility criteria included 18+ years of age, able to speak English, living in a household with at least one smoker and one non-smoker (which could be a child), and allow at least some smoking in the home. Of the 17,138 invited panel members, 5138 (30.0%) started the survey. Of those, 3950 were not eligible, 15 terminated the survey for unspecified reasons, and 599 were eligible but were terminated because the quota for non-smokers had been reached. A total of 250 eligible participants completed the survey in its entirety.

#### Validation sample

Data for the confirmatory factor analysis came from the 6-month follow-up data collection in 2015 from a randomized controlled trial testing the effectiveness of a smoke-free homes (SFH) intervention [22]. Participants were recruited through a 2-1-1 call center (a referral hotline that connects callers to needed social services such as utilities assistance) in Houston, Texas for a SFH intervention trial. Eligibility criteria included being 18 years or older, be able to understand English, having at least one smoker and one non-smoker living in the household, and not having a smoke-free home at baseline. At baseline, 508 participants were randomized into either the intervention or the control group. The intervention group received three mailings and one coaching call to help them create a smoke-free home. The last mailing included a flyer discussing thirdhand smoke. At the second follow-up point, 6 months after baseline, we included the reduced THS scale derived from the exploratory factor analysis from the pilot sample and all 335 participants who were reached answered the relevant questions.

### Measures

#### Pilot sample

##### Beliefs about Thirdhand Smoke scale

Questions for the tested scale were developed after a thorough literature review by an expert panel including tobacco researchers from several universities. Only one item asking about whether breathing air in a room where people smoked previously can harm the health of infants and children was directly retrieved from Winickoff [17]. The remaining 18 items were developed around 6 domains. Table 1 shows the 19 original items and answer choices. THS health impact items were used to ask about general health impact of THS on children [6, 11, 17] and adults [4, 23] as well as cancer specifically [24, 25] with three items. Three items pertaining to THS persistence included days, weeks, and months separately [7, 20]. Three additional items with statements about smoke particles settling with dust [11], accumulating on surfaces [4] and being absorbed into furniture and walls [8] aimed at THS accumulation in the built environment. Four items focused on THS removal with regular/thorough cleaning as well as painting, not being able to completely remove smell from rooms and smoke particles from surfaces, carpet, and walls [8, 26, 27]. Furthermore, three items had statements that addressed THS transmission beyond breathing; these included transmission from skin, hair, and clothing [10, 11] as well as surfaces and children ingesting smoke particles after touching contaminated surfaces. Finally, the last three items focused on THS reduction behaviors: opening windows/using air conditioners; smoking only in the bathroom [28]; and finally having a smoke-free home. Response options were on a 5-point Likert scale from strongly disagree to strongly agree coded as 1 through 5.

**Table 1:** Original and retained items (in bold) of the BATHS scale

The next questions will ask for your opinions on the effects of smoking inside your home.	Strongly disagree	Disagree	Not sure	Agree	Strongly agree
**Breathing air in a room today where people smoked yesterday can harm the health of infants and children.**	1	2	3	4	5
**Breathing air in a room today where people smoked yesterday can harm the health of adults.**	1	2	3	4	5
**Particles in rooms where people smoked yesterday can cause cancer.**	1	2	3	4	5
**Smoke particles can remain in a room for days.**	1	2	3	4	5
**Smoke particles can remain in a room for weeks.**	1	2	3	4	5
Smoke particles can remain in a room for months.	1	2	3	4	5
Cigarette smoke mixes and settles with dust.	1	2	3	4	5
After someone smokes in a room, sticky particles are left on surfaces in the room.	1	2	3	4	5
**Smoke particles get absorbed into furniture and walls.**	1	2	3	4	5
The smell of cigarette smoke can return even after deeply cleaning a smoking room.	1	2	3	4	5
Smoke stains on walls can reappear after walls have been painted.	1	2	3	4	5
Removing smoke particles from carpet is almost impossible.	1	2	3	4	5
Sticky smoke particles cannot be removed from surfaces with regular cleaning.	1	2	3	4	5
**After smoking a cigarette, smoke particles on skin, hair, and clothing can be passed on to others through touch.**	1	2	3	4	5
**After touching surfaces where cigarette smoke has settled, particles can enter the body through the skin.**	1	2	3	4	5
Children who touch surfaces and then put their hands in their mouths can swallow smoke particles.	1	2	3	4	5
**Opening windows or using air conditioners does not eliminate all smoke particles in a room.**	1	2	3	4	5
Smoking only in the bathroom does not stop smoke particles from settling in other rooms.	1	2	3	4	5
Having a smoke-free home will protect nonsmokers from smoke particles in your home.	1	2	3	4	5

##### Smoking and smoke-free home related variables

Participants were asked if they currently smoked cigarettes every day, some days, or not at all. Those reporting smoking every day and some days were coded as current smokers. Smoking ban status in the home was assessed by asking “Which statement best describes the rules about smoking inside your home?” Those responding “There are no rules about smoking inside your home” and “Smoking is allowed anywhere inside your home” were coded as having no ban. Those choosing “Smoking is allowed in some places or at some times” were coded as having a partial ban.

##### Sociodemographics

Participants’ age, gender, race, marital status, education, household income, and home ownership (rent vs. own) were assessed.

#### Validation sample

##### Beliefs about Thirdhand Smoke scale

For the validation sample, we included the nine items from the reduced scale based on the exploratory factor analysis (Table 1 items in bold). Response options were the same as the pilot on a 5-point Likert scale ranging from 1 = Strongly disagree to 5 = Strongly agree. The scale was administered at the second follow-up point, 6 months after baseline.

##### Smoking and smoke-free home related variables

Participants in the validation sample were asked the same questions as those in the pilot sample at six-months post baseline.

##### Sociodemographics

Age, gender, marital status, race, education, employment status, and household income were assessed at baseline.

##### Smoke-free home trial group assignment

Assignment to intervention and control group was random after baseline data collection. This variable was included because intervention participants received educational information regarding THS.

### Data analysis

#### Pilot sample

Descriptive statistics for participant demographics were calculated. Distance of means from the scale mean (3) as well as standard deviations of scale items were assessed. Exploratory factor analysis including scree plots was conducted, and Cronbach’s alpha for the entire scale was calculated. We reduced the scale by eliminating items with the lowest factor loadings step-by-step as long as Cronbach’s alpha was above .90. Multiple Indicators Multiple Causes (MIMIC) models assessing scale invariance by smoking status and home smoking ban status, controlling for age and gender, were conducted as well.

#### Validation sample

Confirmatory factor analysis assessed the fit of the two-factor solution. Descriptive statistics for participant demographics were calculated. Differences between THS total and factor scores by participant characteristics were assessed using t-tests and ANOVAs.

Descriptive and exploratory factor analyses were conducted using SAS 9.4. Confirmatory factor and MIMIC analyses were conducting in MPlus 7.2.

## Results

### Participant characteristics in the pilot sample

Due to the sampling design, 50% of participants were current smokers living with at least one non-smoker, and 50% were current non-smokers living with at least one smoker. Most participants were married or living with a partner (67.2%) and white (78.8%) with at least some college (68.8%) and an annual household income over $25,000 (76.0%).

### Beliefs about Thirdhand Smoke scale development

#### Descriptives of the 19 item scale

Cronbach’s alpha for the entire 19-item scale was 0.95 (both raw and standardized). Exclusion of any item would reduce reliability. Item means were reasonably close to the scale mean and standard deviations were adequate (around 1.0). The scree plot and the eigenvalues were both supporting the conclusion of a two factor solution that explained 97.0% of the variance. Oblique varimax rotation yielded strong factor loadings onto two distinct factors. The first factor can be described as measuring beliefs about THS persistence in the built environment. The second factor includes items that assess beliefs about the impact of THS on health.

#### Scale reduction

Traditional suggestions for scale reduction start with eliminating items with low factor loadings with less than .30 [29]. No items had factor loadings this small. Hence, we sequentially reduced the scale by excluding items with the lowest factor loadings such that the final scale’s reliability as measured with Cronbach’s alpha was greater than 0.90. This approach reduced the scale to nine items: four items related to THS persistence in the environment and five items related to THS impact on health. The final items, their means, standard deviations and factor loadings are presented in Table 2. Factor 1 includes items that describe THS in the built environment, capturing persistence of smoke particles, accumulation of THS, and ineffectiveness of THS reduction by means other than not smoking in the house. Factor 2 includes health impact of THS and transmission of THS through means other than the air. This reduced scale had excellent overall reliability (Cronbach’s alpha = .91) and strong reliability in the sub-scales (Cronbach’s alpha = .88 for both factors).

**Table 2:** Means, standard deviations, and factor loadings of final scale items in the validation sample

Scale item	Mean	(SD)	Factor loadings	
			THS persistence	THS health
Smoke particles can remain in a room for weeks.	3.65	(0.95)	0.759	
Smoke particles can remain in a room for days.	3.89	(0.85)	0.749	
Smoke particles get absorbed into furniture and walls.	4.29	(0.74)	0.582	
Opening windows or using air conditioners does not eliminate all smoke particles in a room.	3.86	(0.87)	0.491	
Breathing air in a room today where people smoked yesterday can harm the health of adults.	4.02	(0.84)		0.721
Particles in rooms where people smoked yesterday can cause cancer.	3.56	(0.98)		0.675
Breathing air in a room today where people smoked yesterday can harm the health of infants and children [17].	4.21	(0.85)		0.576
After smoking a cigarette, smoke particles on skin, hair, and clothing can be passed on to others through touch.	3.67	(0.99)		0.571
After touching surfaces where cigarette smoke has settled, particles can enter the body through the skin.	3.47	(0.87)		0.559

Cronbach’s alpha: .91 for full scale, .88 for each sub-scale

#### Scale invariance and differences in responses

MIMIC models tested indicate scale invariance for smoking status (daily and non-daily smoker versus non-smoker) as well as home smoking ban status (partial versus no ban) making the scale equally suitable for smokers and nonsmokers and those with partial and no smoking ban in their homes. In addition, there were no significant differences in THS beliefs by smoking status or home smoking ban status. However, older participants in the pilot sample were less concerned with the impact of THS on health (*β* = −.009, *p* = .02) indicating a reduction in the THS on health score by .09 for each 10 years increase in age.

### Participant characteristics in the validation sample

Demographics of participants in the validation sample are shown in Table 3. The validation sample participants had a mean age of 41.1 years (*SD* = 12.65), were mostly female (83.6%), African American (69.9%), unemployed (71.6%) and reported an annual household income of $25,000 or less (85.7%) with 46.0% reporting a household income of $10,000 or less. Most participants were renting their home (77.3%) and were smokers themselves (60.3%). About half reported a full smoking ban in their home and an additional 29.0% reported a partial ban.

**Table 3:** Demographic characteristics of study participants in the validation sample and differences in THS overall scores and sub-scale scores

	N	%	THS score		*p*-value	THS persistence		*p*-value	THS health		*p*-value
			Mean	SD		Mean	SD		Mean	SD	
Full sample	335	100	3.92	0.63		3.79	0.66		3.86	0.59	
Age group (N/%)											
18–24	25	7.5%	3.8	0.44	0.68	3.8	0.55	0.58	3.8	0.54	0.77
25–39	124	37.0%	3.8	0.55		3.9	0.59		3.8	0.66	
40–65	174	51.9%	3.9	0.63		4.0	0.67		3.8	0.69	
65 and older	12	3.6%	3.7	0.59		3.8	0.70		3.6	0.62	
Gender (N/%)											
Male	55	16.4%	3.8	0.68	0.46	3.8	0.72	0.2853	3.8	0.75	0.78
Female	280	83.6%	3.9	0.57		3.9	0.62		3.8	0.64	
Marital Status (N/%)											
Married/living with partner	186	55.5%	3.8	0.59	0.49	4.0	0.65	0.48	3.8	0.67	0.58
Single/divorced/separated/widowed	148	44.2%	3.9	0.59		3.9	0.63		3.8	0.65	
Race (N/%)											
White	48	14.3%	3.8	0.56	0.90	4.0	0.61	0.57	3.7	0.60	0.44
African American	234	69.9%	3.9	0.60		3.9	0.64		3.8	0.68	
Latina/o	41	12.2%	3.9	0.58		3.9	0.66		3.9	0.60	
Other	12	3.6%	3.8	0.52		3.8	0.50		3.7	0.69	
Education (N/%)											
High school graduate/GED or less	208	62.1%	3.8	0.59	0.19	3.9	0.65	0.04	3.8	0.65	0.71
Some college/vocational/technical school	109	32.5%	3.9	0.56		4.0	0.59		3.8	0.66	
College graduate or higher	18	5.4%	4.1	0.68		4.3	0.62		3.9	0.79	
Employment (N/%)											
Employed	91	27.2%	3.9	0.54	0.51	3.9	0.61	0.93	3.9	0.61	0.21
Not employed	240	71.6%	3.8	0.61		3.9	0.64		3.8	0.68	
Income (N/%)											
$10,000 or less	154	46.0%	3.8	0.57	0.91	3.9	0.62	0.61	3.8	0.65	0.99
$10,001 to $25,000	133	39.7%	3.9	0.62		4.0	0.66		3.8	0.68	
$25,001 to $50,000	35	10.4%	3.8	0.61		3.9	0.62		3.8	0.69	
$50,001 to $75,000	6	1.8%	3.9	0.58		4.1	0.70		3.7	0.55	
More than $75,000	4	1.2%	3.9	0.17		4.1	0.55		3.8	0.25	
Home ownership											
Owner	70	20.9%	4.0	0.62	0.04	4.1	0.66	0.02	3.9	0.68	0.13
Resident	259	77.3%	3.8	0.58		3.9	0.62		3.8	0.68	
Home smoking ban											
No ban	70	20.9%	3.6	0.60	0.001	3.8	0.62	0.02	3.5	0.69	0.0004
Partial ban	97	29.0%	3.8	0.58		3.9	0.63		3.8	0.65	
Full ban	168	50.1%	4.0	0.57		4.0	0.64		3.9	0.63	
Smoking status											
Smoker	202	60.3%	3.8	0.59	0.02	3.9	0.64	0.09	3.7	0.65	0.01
Non-smoker	133	39.7%	4.0	0.58		4.0	0.62		3.9	0.66	
Group assignment											
Intervention group	158	47.2%	3.97	0.59		4.04	0.66		3.89	0.65	
Control group	177	52.8%	3.76	0.57	0.001	3.82	0.59	0.002	3.69	0.66	0.005

### Beliefs about Thirdhand Smoke scale psychometric properties

Confirmatory factor analysis showed the good model fit for the two factor model (*Χ* ^2^(24) = 65.546, *p* < .0001, RMSEA = 0.072, 90%CI (RMSEA) = [0.051; 0.093], CFI = 0.957, TLI = 0.936, SRMR = 0.048). Error covariances between items about THS being harmful to children and to adults were allowed to covary as were items asking about smoke particle transfer through touch and smoke particles entering the body through the skin. Both adjustments improved the a-priori two factor model without error covariances constrained (*Χ* ^2^(26) = 140.086, *p* < .0001, RMSEA = 0.114, 90%CI(RMSEA) = [0.096; 0.133], CFI = 0.883, TLI = 0.838, SRMR = 0.061). The a-priori specified model had statistically significantly better fit than a one factor model (ΔΧ^2^(1) = 38.532, *p* < .0001). The high correlation between the factors of 0.82 might indicate low convergent validity. However, no factor loadings were weak (i.e. below .40) and most were strong (i.e. greater than .60).

The factor scores as well as the total scale score differed significantly by key participant criteria indicating good construct validity. Those who were in the intervention group and thus were exposed to educational materials on THS scored significantly higher on the total THS scale score as well as the sub-scores than participants in the control group (*p* = .0001 for the full score, *p* = .002 for THS persistence score, *p* = .005 for THS health score). Furthermore, a similar pattern was observed when comparing those who had a full smoking ban in their home compared to those who had a partial or no ban (*p* = .001, *p* = .002, *p* = .001, respectively). Participants who owned their homes had higher THS persistence (*p* = .04) and total scores (*p* = .02), but had comparable scores on THS health. Smokers had, on average, lower total THS scores (*p* = .02) and lower THS health scores (*p* = .01). However, there was no statistically significant difference between smokers and non-smokers on the THS persistence score. When looking at demographics, the only differences found in THS scores were by educational attainment where those with more education had higher THS persistence scores (*p* = .04). There were no significant differences in any of the scores by age, gender, race, marital status, income, and employment status.

## Discussion

While prior research [17, 30–32] has used one specific item to measure this construct, the current study is the first to our knowledge to develop a valid and reliable measure of beliefs regarding THS. This is a critical step in research aimed at addressing THS exposure. As smoke-free air policies are more commonly being implemented both in public and in private spaces [17, 31, 32], individual understanding of THS and its impact will be critical in further reducing the harm of tobacco smoke. In particular, intervening on beliefs about THS may prove to be beneficial in reducing THS and SHS exposure. Consequently, the creation of a valid and reliable scale of beliefs regarding THS is critical in assessing these intervention efforts.

This study yielded a valid and reliable 9-item scale assessing this construct, reduced from originally 19 items. The final scale with scoring instructions is available in the Additional file 1. While we had conceptualized five domains, the exploratory factor analysis of the full scale indicates that there are only two distinct domains measured by these 19 items. Interestingly, the two factors that emerged related to: 1) how THS operates within the built environment, i.e. the persistence of smoking particles in indoor spaces, and 2) the impact of THS on health. While the former subscale may demonstrate how THS functions within the concrete setting in which smoking occurs, the second measures perceived risk to human health, which is an important theoretical construct related to smoking behavior [33]. In the context of multiunit housing managers or owners, the former may be of particular interest, as they consider the impact of smoking on rental cleanup/deposits or property values [34], and might not be concerned with the negative health effects of smoking on renters.

The scale scores differed, as expected, by smoking status and smoke-free ban status in the home. In addition, we found that those who owned their home scored higher on the THS persistence sub-scale. This might be due to home owners living longer in their home than those who rent and thus observing the persistence of the smell and discoloration of walls or due to homeowners increased interest in preserving property values. However, future studies might explore reasons for those differences. In addition, the intervention participants had higher THS sub- and total scores. Thus, having exposure to educational materials may create awareness of THS, its persistence in the environment and its harms.

There are limitations to the current study. While participants for the pilot study were recruited from across the United States, the validation sample was limited in location to Houston, TX. Thus, findings cannot be extended to those living in different situations. Moreover, this scale should be tested in different populations in varied geographic locations and in those with higher income to explore its external validity. Future studies should test a larger number of possible validity variables. Future research examining how this scale operates alongside measures of perceived risk or harm of smoking, SHS, and receptivity to policies to reduce tobacco smoke exposure could enhance our understanding of the validity of this measure.

## Conclusions

This study has important implications for research and practice. Intervention research that focuses on reducing the harms of tobacco smoke exposure should focus both on the harms/effects of SHS and THS as intervention messages. Therefore, a reliable scale of THS beliefs may be a proxy measure of propensity to lower SHS and THS exposure in homes or other personal spaces such as cars. In addition, examining results across different populations may be helpful in identifying high-risk groups for increased THS exposure and groups more likely to be responsive to interventions that emphasize harm of THS. Practitioners can use the scale to conceptualize intervention experiences to increase understanding of how THS operates within smoke-free homes initiatives that would reduce carcinogens in environments where smoking either still occurs or SHS incursion is a problem.

## Additional file

Additional file 1: Beliefs About ThirdHand Smoke (BATHS)© scale. (PDF 429 kb)

## Abbreviations

BATHS: Beliefs About ThirdHand Smoke
MIMIC: Multiple causes multiple indicators
SFH: Smokefree home
SHS: Secondhand smoke
THS: Thirdhand smoke
