# Supplementary material for: Development of a scale assessing Beliefs About ThirdHand Smoke (BATHS)
Source: Tob Induc Dis. 2017 Jan 17;15:4. doi: 10.1186/s12971-017-0112-4 (PMC5240270; doi:10.1186/s12971-017-0112-4)
Supplement: Additional file 1: — Beliefs About ThirdHand Smoke (BATHS)© scale. (PDF 429 kb) [file 12971_2017_112_MOESM1_ESM.pdf]

Beliefs About ThirdHand Smoke (BATHS)© scale

Regine Haardörfer, Carla J. Berg, Cam Escoffery, Łucja T. Bundy, Melbourne Hovell, Michelle C. Kegler

The following questions ask for your opinions on the effects of smoking inside your home.

	Strongly disagree	Disagree	Not sure	Agree	Strongly agree
1. Breathing air in a room today where people smoked yesterday can harm the health of infants and children.	1	2	3	4	5
2. Breathing air in a room today where people smoked yesterday can harm the health of adults.	1	2	3	4	5
3. Particles in rooms where people smoked yesterday can cause cancer.	1	2	3	4	5
4. Smoke particles can remain in a room for days.	1	2	3	4	5
5. Smoke particles can remain in a room for weeks.	1	2	3	4	5
6. Smoke particles get absorbed into furniture and walls.	1	2	3	4	5
7. After smoking a cigarette, smoke particles on skin, hair, and clothing can be passed on to others through touch.	1	2	3	4	5
8. After touching surfaces where cigarette smoke has settled, particles can enter the body through the skin.	1	2	3	4	5
9. Opening windows or using air conditioners does not eliminate all smoke particles in a room.	1	2	3	4	5

To derive sub-scores for the BATHS scale, calculate

1. The mean of items 1,2,3,7, and 8 for the THS impact on health sub-scale
2. The mean of items 4,5,6, and 9 for the THS persistence in the environment sub-scale

© Emory Prevention Research Center
